# A computational study of VEGF production by patterned retinal epithelial cell colonies as a model for neovascular macular degeneration

**DOI:** 10.1186/s13036-017-0063-6

**Published:** 2017-08-02

**Authors:** Qanita Bani Baker, Gregory J. Podgorski, Elizabeth Vargis, Nicholas S. Flann

**Affiliations:** 1 Jordan University of Science and Technology, Irbid, Jordan; 2 Biology Department, Utah State University, Logan, 84322 USA; 3 Center for Integrated BioSystems, Utah State University, Logan, 84322 USA; 4 Biological Engineering Department, Utah State University, Logan, 84322 USA; 5 Synthetic Biomanufacturing Institute, Logan, 84322 USA; 6 Institute for Systems Biology, Seattle, 98109 USA; 7 Computer Science Department, Utah State University, Logan, 84335 USA

**Keywords:** Micropatterning, Auto-regulation, Vascular endothelial growth factor, Age-related macular degeneration, Retinal pigment epithelial cells, Age-related macular degeneration

## Abstract

**Background:**

The configuration of necrotic areas within the retinal pigmented epithelium is an important element in the progression of age-related macular degeneration (AMD). In the exudative (wet) and non-exudative (dry) forms of the disease, retinal pigment epithelial (RPE) cells respond to adjacent atrophied regions by secreting vascular endothelial growth factor (VEGF) that in turn recruits new blood vessels which lead to a further reduction in retinal function and vision. In vitro models exist for studying VEGF expression in wet AMD (Vargis et al., Biomaterials 35(13):3999–4004, 2014), but are limited in the patterns of necrotic and intact RPE epithelium they can produce and in their ability to finely resolve VEGF expression dynamics.

**Results:**

In this work, an in silico hybrid agent-based model was developed and validated using the results of this cell culture model of VEGF expression in AMD. The computational model was used to extend the cell culture investigation to explore the dynamics of VEGF expression in different sized patches of RPE cells and the role of negative feedback in VEGF expression. Results of the simulation and the cell culture studies were in excellent qualitative agreement, and close quantitative agreement.

**Conclusions:**

The model indicated that the configuration of necrotic and RPE cell-containing regions have a major impact on VEGF expression dynamics and made precise predictions of VEGF expression dynamics by groups of RPE cells of various sizes and configurations. Coupled with biological studies, this model may give insights into key molecular mechanisms of AMD progression and open routes to more effective treatments.

## Background

Age-related macular degeneration (AMD) is a leading cause of irreversible blindness, particularly among adults over the age of 50 [2–4]. In AMD, degeneration of retinal pigment epithelial (RPE) cells, a type of neural cell that provides metabolic support to photoreceptor cells, severely damages vision. There are two forms of AMD, one involving acellular debris (dry AMD), and the other involving neovascularization of the retina from the underlying choriocapillaris (wet AMD) [5].

Cell culture models that control the spatial organization and growth of RPE cells can provide valuable tools for understanding cell behavior in AMD and its interaction with vascular endothelial growth factor (VEGF). VEGF is the primary signaling molecule that stimulates angiogenesis [6] and is an important biomarker of AMD [7]. In the retina, VEGF is secreted in the RPE and is the primary driver of retinal vasculature development [8, 9]. Monitoring the expression of VEGF within controlled environments of model systems can lead to new insights that improve our understanding of the initiation and progression of AMD.

**Fig. 1:**
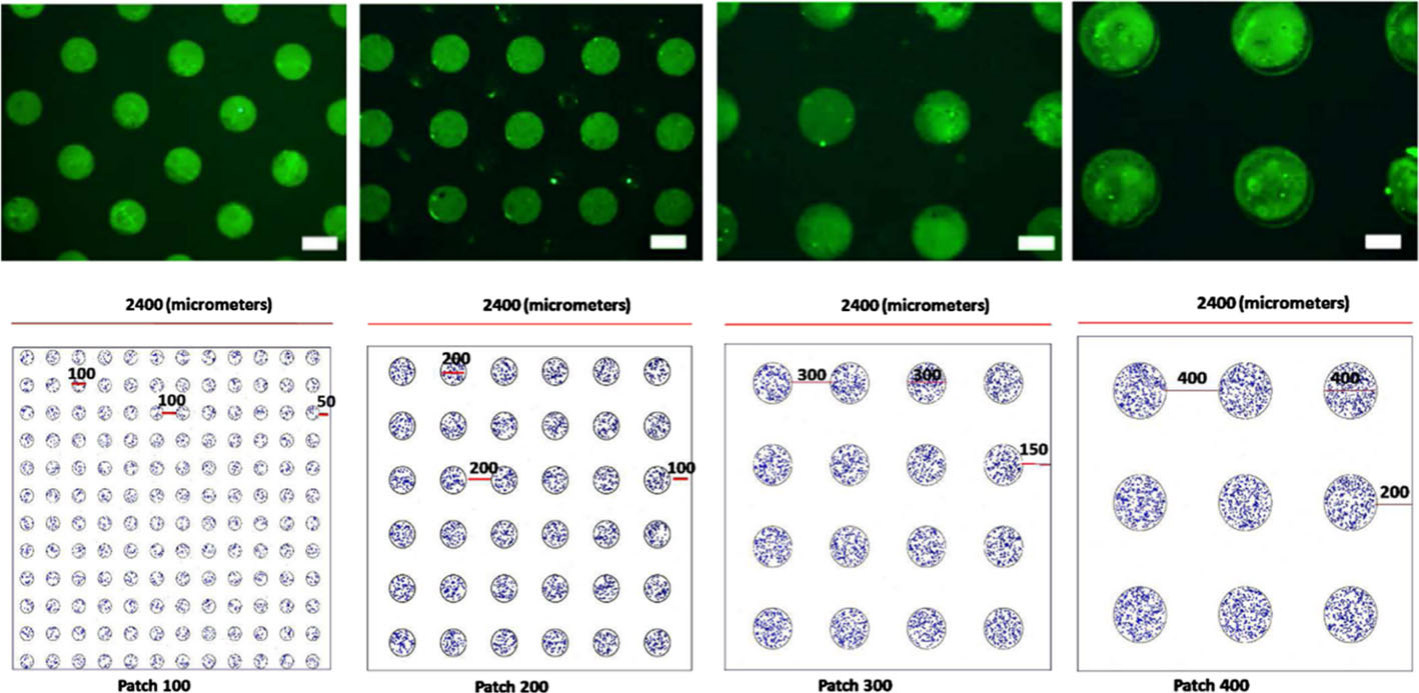
Patch configurations. The *top* row shows cropped images of the experimental patches of fluorescent fibronectin that was used to form the patches for cell growth. The *white scale bars* are 100*μ*m. The *bottom* images show the simulated configurations of the cells (the *blue circles*). In all cases, patches occupied the same total area (1.131 mm^2^) that comprised 20% of the total simulation domain (2.4 mm × 2.4 mm.)

In the cell culture model of AMD that is simulated in this study, micropatterning techniques are used to restrict the location and shape of the substrate on which cells can attach and grow [10–12]. The impact of micropatterning on cellular functions and morphologies has been investigated with many types of cells including fibroblasts [13], neuronal cells [14], stem cells [15], epithelial cells [16], cancer cells [17], and retinal pigment epithelial cells [1, 18–21]. Vargis et al. [1] used micropatterned surfaces to control the spatial organization of RPE cells to explore how atrophy or tissue damage within the retina affect VEGF production (Fig. 1). While cell culture provides a model for replicating disease states associated with the deterioration of retinal tissue during AMD, the stimuli leading to enhanced VEGF secretion from RPE cells and the subsequent neovascularization of the choroid are still not fully understood [22, 23], and little is known about how VEGF production is regulated in the eye [9]. In addition, much remains to be learned about how anti-angiogenic drugs work in the retina [24], how and why AMD and other retinal diseases become resistant to treatment, and the types of patients that can benefit most from anti-angiogenic drugs [25]. Computational approaches combined with experimental studies have the potential to shed light on these issues by providing a platform for generating and testing hypotheses related to the regulation of VEGF production and transport in the retina [26].

Developing an *in silico* framework for the cell culture micropatterning AMD model provides a beneficial system for evaluating the spatiotemporal effects of VEGF transport and expression within these controlled environments and in replicating the pathology of AMD to gain new insights on disease progression and outcomes. In silico models can also be used to study internal and external regulatory mechanisms influenced by feedback from the evolving cellular environment. Developing these predictive models is essential to identify biological pathways that may be targeted by new pharmaceutical agents.

The goal of this study was to develop an in silico model to replicate and extend the cell microprinting model for AMD reported in [1]. The in silico model employs a two dimensional representation of the cellular culture because in the microprinting model, a monolayer of RPE cells form on the printed disks. While a two dimensional model is sufficient to replicate this bioengineered study, more realistic models that incorporate photoreceptors, and bipolar, amacrine, ganglion cells would require three dimensions.

Using this computational model, we studied the growth of RPE cells in discrete patches of different sizes and configurations to learn how cell arrangements can effect VEGF expression. The level of VEGF in each group of cells was studied as a function of cell number and patch area over time. To explore the hypothesis that VEGF expression is linked to global VEGF concentration, VEGF expression from various sized patches was quantified following VEGF administration. This study complements experiments using cell culture and provides a framework that can be used to investigate the influence of cell patterning on the secretion of VEGF by the RPE and opens a path towards mimicking the effects of tissue damage. This model extended the study of Vargis *et al* [1] and made predictions about VEGF regulation and expression in cell configurations that could not have been produced experimentally. The in silico model has the potential to examine the effects of anti-VEGF agents that may aid in the optimization of anti-angiogenic therapeutics and to be extended to other disorders that involve localized cell death within an epithelium.

## Methods

### Hybrid agent-based model framework

The agent-based modeling framework known as iDynoMiCs [27] was extended to simulate the effect of RPE cell distribution on VEGF expression. This modeling framework consists of discrete and continuous elements, making this a hybrid model. The discrete elements are particles each representing an individual cell. Particles mechanically interact with one another and secrete, consume or react to soluble molecules. They are positioned in space and occupy the volume of a single cell. The continuous elements of the model are a collection of soluble molecules (referred to as solutes) that could include nutrients, oxygen, and signaling molecules such as VEGF. A set of partial differential equations (PDEs) defines the interactions of molecules with cells and each other as they diffuse and participate in a variety of reactions.

Reactions between solutes and particles drive particle growth. As the mass of a particle increases, so does its radius. When the radius equals or exceeds a maximum state-specific particle size (for RPE cells, *r*_*sp*_ is defined in Table 1), the particle is divided in two along a random cleavage plane, such that the sum of the volumes of the new spherical particles approximately equals the specified maximum volume. The two smaller particles are positioned without mutual overlap in the place of the parent particle.

**Table 1:** Model’s parameter descriptions

Parameter	Value	Units	Description	Ref
*D*_*V*_	5.8×10^−11^	m^2^/s	Diffusion coefficient	[28]
*α*_*M*_	1.0194	1/h	Max. growth rate for RPE cells	[29]
*k*	0.13	pg/ml	Half saturation rate	[30]
*α*_*V*_	0.09	1/h	Max. VEGF secretion rate	[30]
*β*	0.850	unitless	Binding affinity	[30]
*r*_*sp*_	6.2996	*μ*m	Cell division radius	[1]

As the simulation proceeds, particles move because of growth and division, and solute distributions change due to reactions and diffusion. In a single simulation step, first the biomechanical forces arising from growth are relaxed by moving particles to avoid overlap [27], then a PDE solver is applied to resolve all the local changes in solute concentrations. The solute fields are kept in steady-state with respect to the particle movement because reactions and diffusion occur much more rapidly (on the order of seconds or minutes) than changes in cell positions (on the order of hours to days).

Cyclic boundary conditions are used for the sides of the 2D simulation domains illustrated in Fig. 1. This boundary type allows the representation of larger domains by assuming that the computation domain is replicated indefinitely. Solute concentrations are kept constant across cyclic boundaries, and any particle crossing one of the cyclic boundaries is instantly moved to the connected boundary.

### Modeling different RPE configurations

The distributions of cells studied by Vargis et al. were replicated as shown in Fig. 1. Patch diameters were 100, 200, 300 and 400 *μ* *m*. In these configurations, the total area of the domain was 5.76 mm^2^ and the area occupied by patches that could support the growth of cells was constant across all simulations and equal to 1.13 mm^2^ or 20% of the simulation domain.

The number of cells in each patch at the beginning of the simulations is given in Table 2. The doubling time was 36 h, and the simulations ran for 72 h.

**Table 2:** Initial number of cells in each patch. These values match those used in the Vargis et al. study [1]

PatchSize (*μ* *m*)	Initial number of cells.
100	15
200	74
300	189
400	351

The VEGF distribution is determined by diffusion through the medium, secretion as a function of particle mass *M* and local VEGF concentration *V* as given by Eq. 1. The diffusion coefficient of VEGF (*D*_*V*_) is set to 5.8×10^−11^ *m* ^2^/*s*, from experiments described in [28]. The first term of Eq. 1 accounts for VEGF concentration changes due to diffusion, and the second term accounts for the auto-regulation of VEGF through negative feedback.

1 $$ \frac{\partial V}{\partial t}=D_{V}\bigtriangledown^{2} V + \alpha_{V} \frac{k}{ \beta V+k} \: M   $$

Vargis et al. [1] studied the effect negative feedback of VEGF on its own production by adding a form of VEGF that was not detected in their VEGF assay (denoted as *V* *a*). The extended model is given in Eq. 2.

2 $$ \frac{\partial V}{\partial t}=D_{V}\bigtriangledown^{2}V + \alpha_{V} \frac{k}{ \beta(V+ V\!a)+k}\: M   $$

The growth rate of RPE cells is given by Eq. 3. This equation assumes that cell growth is exponential throughout the simulation, a reasonable assumption given the low initial cell densities in each patch and the limited amount of time (2 cell doublings over 72 h) over which the simulation runs.

3 $$ \frac{\partial M}{\partial t}= \alpha_{M}\: M \dot{}  $$

Table 1 summarizes the parameters and their values used in the equations above.

## Results

### VEGF expression

**Fig. 2:**
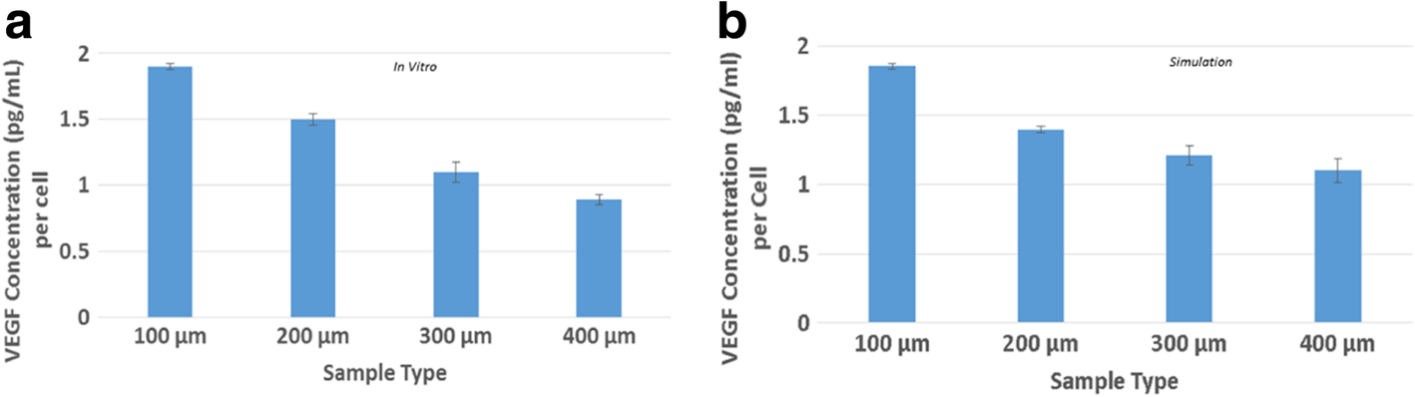
VEGF expression is given as the concentration of VEGF per cell at the end of the cell culture study of Vargis et al. [1] (*open bars*) and the end of the 72 h simulation (*closed bars*). Error bars show one SD. **a** Experimental results, **b** Simulated results

The amount of VEGF expressed per cell was calculated by determining the total amount of VEGF produced over the course of each simulation and dividing this by the final number of cells. Figure 2 presents the simulated values shown alongside those measured in cell culture by Vargis et al. [1]. The agreement is excellent, with the results showing an inverse relationship between VEGF concentration per cell and patch size. This observation was hypothesized in [1] to be due to the locally higher VEGF concentrations experienced by cells in larger patches resulting in lower VEGF expression per cell because of negative feedback. The fact that the simulation captured this effect qualitatively and was able to accurately quantify it provides support for the model.

**Fig. 3:**
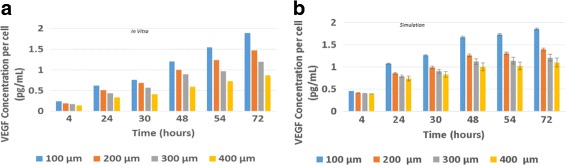
Time course of VEGF expression measured in cell culture (*panel a*) and predicted by the model (*panel b*); Error bars show one SD). **a** Experimental results, **b** Simulated results

The performance of the model in simulating the time course of VEGF expression over the model run is shown in Fig. 3. The qualitative agreement is excellent. In this case, the amount of VEGF produced per cell increases in each configuration of cells over the course of both the cell culture study (panel a) and the simulation (panel b). Notably, the model once again captured the inverse relationship between patch size and VEGF production across all time points. The quantitative agreement is strong particularly at the end of the simulation (for example, note the virtually identical VEGF expression in cells in 100 mm patches at 72 h in the cell culture and simulation studies) and less so at times less than 72 h. For these earlier times, the model predicts higher VEGF expression than measured in the cell culture study. However, even at these time points, the agreement between the experimentally measured and predicted VEGF levels differs at most by a factor of 1.3 (this is for the predicted/observed VEGF values in 100 *μ*m patches at 48 h). In short, the model can predict the qualitative trends accurately and can make quantitative predictions that differ by less than 50% from measured values.

### Exploration of autoregulation of VEGF expression

We hypothesized that there would be higher levels of VEGF per cell expressed in small patches because these cells experienced lower initial levels of VEGF than did the cells in larger patches. Although cells were plated at the same cell density in all patch configurations, cells in the smallest patches have a greater chance of being at the edge of a patch rather than surrounded by neighboring cells. This edge effect would lead to a lower average VEGF concentration around each cell. Given the negative feedback loop that regulates VEGF expression, cells in smaller patches are predicted to produce more VEGF. A prediction of this hypothesis is that adding VEGF early in the model run will suppress VEGF production, particularly from cells in the smallest patches. This prediction was tested and shown to be correct in the cell culture system. Experimentally, this was accomplished by the addition a form of VEGF (represented as *V* *a*) that binds with equal affinity to the RPE receptors, but can be distinguished from the VEGF produced by cells at assay [1]. In a simulation, this effect of the added VEGF is represented in Eq. 2.

**Fig. 4:**
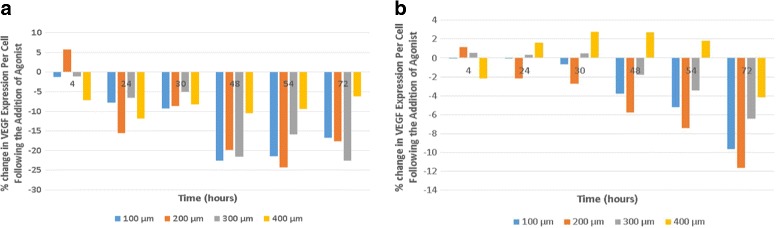
Effect of VEGF addition on the VEGF production [1]. The VEGF agonist (*V* *a*), not detectable in the VEGF assays, was added 20 h after the plating the cells. **a** Experimental results, **b** Simulated results

In the simulation and cell culture studies, VEGF was added at a concentration of 5 ng/ml 20 h after the initial cell seeding. This VEGF concentration is roughly 5-fold higher than the maximum VEGF levels produced after 72 h of in vitro cell culture. A comparison of the simulated and experimentally determined results is shown in Fig. 4. These results are expressed as the percentage change in VEGF produced per cell relative to the control without added VEGF.

As for previous results, the simulated and experimental results are in qualitative agreement. Cells in smaller patches on average did respond more strongly than cells in larger patches to the added VEGF. However, on average the model predicted about a 2-fold lower response to added VEGF in the medium relative to the experimental observations. A possible explanation for this discrepancy is that the values for VEGF binding affinity *β* and half saturation *k*, two key parameters that control negative feedback for VEGF production (see Eq. 2), are estimated from a computational study [30]. Slight discrepancies between the estimated and actual, but unknown, values of *β* and *k* could easily lead to the mismatch between experimental and simulated values.

In both the cell culture investigation and the simulation, the VEGF levels per cell obtained from smaller patch sizes (100, 200, 300 *μ*m) decreased after the VEGF was added. This result supports the hypothesis that cells within these smaller patches reduce VEGF expression levels because of the increased levels of VEGF within their local environment. Cells in patches of larger sizes (400 *μ*m) already encountered higher levels of VEGF. Therefore, they showed smaller changes in VEGF expression levels after VEGF addition.

### Using the Model to Extend Experimental Observations - VEGF Distributions

**Fig. 5:**
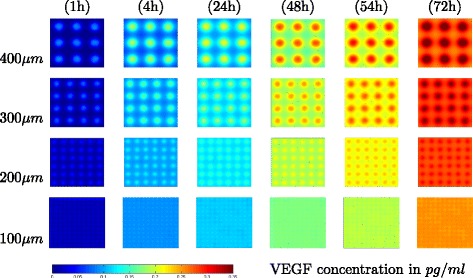
Distributions of VEGF over the course of model runs. Rows are different patch sizes, columns are time points. All figures are colored using the same scale and so may be compared directly

Having established the utility of the model, we applied it to extend experimental observations of VEGF distributions that are important in shaping tissue responses to VEGF but cannot be studied using current experimental methods. Figure 5 shows the predicted VEGF distribution profiles over the course of model runs with different patch sizes. These distributions are due purely to VEGF diffusion and metabolism and do not account for any circulation of VEGF. The results are striking in at least two ways. First, they show that the predicted VEGF distribution is much more uniform across the simulation domain for small patches than for large patches. Next, they support the idea that cells in small patches experience a much lower average VEGF concentration than cells in larger patches, particularly at earlier times. This observation supports the negative-feedback hypothesis for why cells in smaller patches are expected to express more VEGF per cell than cells in larger patches. Take as a whole, these predictions of VEGF distributions in different cellular configurations highlight the importance of the geometry and dimensions of damaged and undamaged tissue in AMD and other disorders that involve necrosis.

### Using the Model to Study the Effect of New Cellular Configurations

**Fig. 6:**
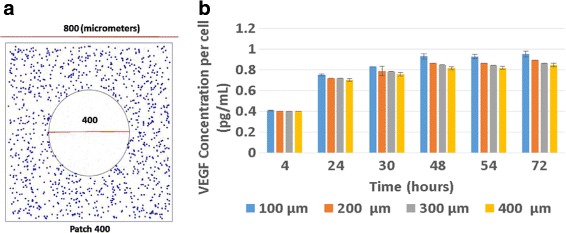
Prediction of VEGF produced by an inverted pattern of cell-containing and cell-free zones. **a** The inverse pattern illustrated for one cell-free region of diameter 400 *μ*m. **b** VEGF expression per cell in inverted cell configurations. In these cases, the numbers refer to the diameters of cell-free circles arranged in the same pattern as in Fig. 1

Current micropatterning technologies allow printing uniform circular domains for cell growth on a tissue culture plate but do not easily allow printing the inverse pattern of open circular spaces within a field of cells. Unfortunately, this latter arrangement is a more realistic model of the necrotic retinal lesions seen in AMD. The benefit of a model is its ability to rapidly test cell configurations that are difficult or impossible to explore experimentally. We did this by modeling the inverse pattern of the pattern studied by Vargis et al. [1]. This inverted pattern is illustrated in Fig. 6a.

The predicted VEGF expression per cell in these inverted patterns of cell-free circles of different sizes are shown in Fig. 6b. The notable findings are that the differences between patch sizes are virtually eliminated and that the amount of VEGF expressed per cell is reduced to roughly half of that seen in the standard pattern of cell-filled circles. Both results are likely due to the larger cell-filled area in the inverted configuration (20% cell-filled area in each standard configuration and 80% cell-filled area in each inverted configuration). This pattern reduces edge effects, is predicted to result in higher local VEGF levels. In turn, this is predicted to reduce VEGF expression through negative feedback.

## Discussion

The model was used to provide insights into molecular events that are not accessible using current experimental techniques. Here, the model predicted that VEGF would be present at lower levels and be more evenly distributed when cells were configured in many small patches than in fewer large patches. These predicted VEGF distributions are consistent with both the experimentally determined and model-based results that VEGF expression per cell is strongest in cells distributed in small patches. These results are significant in understanding how different patterns of retinal necrosis may affect neovascularizationin AMD.

The model was applied to predict how a cellular configuration that cannot be easily designed in the laboratory will influence VEGF expression. In this configuration, a regular grid of open circles without cells is embedded in a surface with full cell coverage. This pattern represents an inversion of the standard tissue-print model of AMD and more closely resembles necrotic lesions within the retinal epithelium. The model predicted that VEGF production is nearly invariant with respect to the size of the open circles. Importantly, this result demonstrates that without significant empty space bordering the fields of cells, negative feedback predominates, leading to low, constant VEGF production independent of the size of cell-free zones.

A next step will be to extend these studies to different tissue configurations, including those that more closely match the diseased retina in AMD, and to consideration of additional parameters, such as oxidative stress [31] and the effects of inflammatory cytokines [32], that are important in the development and progression of AMD. Understanding how different patterns of necrosis can disrupt VEGF signaling will be important for developing rational therapies of neovascular AMD. Pairing cell culture studies that use micropatterning and precise measures of VEGF expression with model-based approaches offers a promising route toward accomplishing this goal.

## Conclusions

Cell culture provides a model for replicating disease states associated with the deterioration of retinal tissue during AMD, the stimuli leading to enhanced VEGF secretion from RPE cells and the subsequent neovascularization of the choroid are still not fully understood [22, 23], and little is known about how VEGF production is regulated in the eye [9].

This study presents a hybrid agent-based model to support and extend cell culture models of AMD. The modeling framework was validated using experimentally gathered data on VEGF expression by RPE cells micropatterned in tissue culture dishes [1]. Simulated results were in excellent agreement with the qualitative findings of Vargis et al. [1] and overall were in good quantitative agreement regarding the amount of VEGF expressed per cell in different patterning configurations.

## Abbreviations

AMD: Age-related macular degeneration
PDE: Partial differential equations
RPE: Retinal pigment epithelial cells
VEGF: Vascular endothelial growth factor
